# MiR-200b attenuates IL-6 production through IKKβ and ZEB1 in human gingival fibroblasts

**DOI:** 10.1007/s00011-018-1192-1

**Published:** 2018-10-10

**Authors:** Sari Matsui, Liming Zhou, Yohei Nakayama, Masaru Mezawa, Ayako Kato, Naoto Suzuki, Natsuko Tanabe, Tomohiro Nakayama, Yuki Suzuki, Noriaki Kamio, Hideki Takai, Yorimasa Ogata

**Affiliations:** 1 Department of Periodontology, Nihon University School of Dentistry at Matsudo, Matsudo, Chiba 271-8587 Japan; 2 Stomatological Hospital of Anhui Medical University, Hefei, Anhui China; 3 Research Institute of Oral Science, Nihon University School of Dentistry at Matsudo, Matsudo, Chiba 271-8587 Japan; 4 Department of Biochemistry, Nihon University School of Dentistry, Chiyoda-ku, Tokyo 101-8310 Japan; 5 Laboratory of Veterinary Radiology, Nihon University College of Bioresource Sciences, Fujisawa, Kanagawa 252-0880 Japan; 6 Department of Preventive Veterinary Medicine and Animal Health, Nihon University College of Bioresource Sciences, Fujisawa, Kanagawa 252-0880 Japan; 7 Department of Microbiology, Nihon University School of Dentistry, Chiyoda-ku, Tokyo 101-8310 Japan

**Keywords:** Human gingival fibroblasts, IKKβ, IL-6, miR-200b, Periodontitis, ZEB1

## Abstract

**Objective:**

MicroRNAs (miRNAs) play important roles in biological processes such as cell differentiation, development, infection, immune response, inflammation and tumorigenesis. We previously reported that the expression of miR-200b was significantly increased in inflamed gingiva compared with non-inflamed gingiva. To elucidate the roles of miR-200b in the inflamed gingiva, we have analyzed the effects of miR-200b on the expression of IL-6 in human gingival fibroblasts (HGF).

**Materials and methods:**

Total RNA and protein were extracted from HGF after stimulation by interleukin-1β (IL-1β; 1 ng/ml) or tumor necrosis factor-α (TNF-α; 10 ng/ml) and transfected with miR-200b expression plasmid or miR-200b inhibitor. IL-6, IL-1β, inhibitor of nuclear factor kappa-B kinaseβ (IKKβ), Zinc-finger E-box-binding homeobox 1 (ZEB1) and E-cadherin mRNA and protein levels were analyzed by real-time PCR and Western blot.

**Results:**

IL-1β and TNF-α increased IL-6 mRNA and protein levels, and they were significantly suppressed by miR-200b overexpression, whereas they were further increased by miR-200b inhibitor in HGF. IKKβ and ZEB1 which are target genes of miR-200b negatively regulate E-cadherin. MiR-200b suppressed the expression of IKKβ and ZEB1 and increased E-cadherin mRNA and protein levels in HGF.

**Conclusions:**

These results suggest that miR-200b attenuates inflammatory response via IKKβ and ZEB1 in periodontal tissue.

## Introduction

MicroRNAs (miRNAs) are single-stranded, small non-coding RNA molecules regulating gene expression by hybridization to targeted transcripts. They play key roles on biological processes such as cell differentiation, development, infection, immune response, inflammation and tumorigenesis. Therefore, the role of miRNAs has been extensively studied in the regulation of cellular processes, including proliferation and differentiation, apoptosis, cancer and viral infections. Several studies have reported that the functions of miRNAs are associated with the regulation of inflammatory responses [1–3]. Recent studies have demonstrated the differences in miRNA expression between inflamed and healthy tissues [4–6]. We have previously demonstrated that the three most overexpressed miRNAs in inflamed gingiva from Japanese chronic periodontitis patients were miR-150, miR-223 and miR-200b, and inflammatory cytokines induced miR-150 and miR-223 expressions in human gingival fibroblasts (HGF) [6, 7]. MiR-223 increased the inflammation response via inhibitor of nuclear factor kappa-B (IκB) kinase α (IKKα) and mitogen-activated protein kinase phosphatase-5 (MKP-5) [7], whereas miR-200b suppressed tumor necrosis factor-α (TNF-α) induced interleukin-8 (IL-8) secretion and tight junction disruption of intestinal epithelial cells [8]. miR-200a/200b inhibited high-glucose induced endothelial inflammation by regulating *O*-GlicNAc transferase-mediated protein O-GlcNAcylation [9]. miR-200b/c attenuated lipopolysaccharide-induced early pulmonary fibrosis by targeting Zinc-finger E-box-binding homeobox 1/2 (ZEB1/2) via p38 mitogen-activated protein kinase (MAPK) and transforming growth factor-β (TGF-β)/smad3 signaling pathways [10].

Periodontal disease is a chronic inflammatory disease caused by periodontopathic bacteria, such as *Porphyromonas gingivalis* and viruses, which lead to inflammation, destruction of periodontium and tooth loss [11–14]. The response to periodontal pathogens is determined by both innate and adaptive immune responses. The innate immune response plays a critical role in defense against putative periodontal pathogens and virulence factors [14]. Cytokines are central regulators of the immune response which are produced by various cell types including epithelial cells, fibroblasts, dendritic cells, macrophages and T-helper cells in response to microbes [15]. Inflammatory cytokines are regulated by activation of nuclear factor-kappa-light-chain-enhancer of activated B cells (NF-κB), interferon regulatory factor (IRF) family of transcription factors and MAPKs [16, 17]. NF-κB consists of five family members, including RelA (p65), RelB, c-Rel, NFκB1 (p105/p50) and NFκB2 (p100/p52) that play critical roles in inflammation, immunity, differentiation, cell proliferation and apoptosis. NF-κB exists as a heterodimer consisting of p65 and p50 subunits which are associated with IκB in the cytoplasm as an inactive form. IκB is phosphorylated by IKK and NF-κB is activated by the phosphorylated IκB and induces transcription of a variety of target genes, including L-1β and IL-6 [18–20]. The IKK complex is composed of three subunits each encoded by a separate gene, such as IKKα [also known as conserved helix-loop-helix ubiquitous kinase (CHUK)], IKKβ [also known as inhibitor of kappa light polypeptide gene enhancer in B-cells, kinase beta (IKBKB)] and IKKγ [also known as NF-kappa-B essential modulator (NEMO) or inhibitor of nuclear factor kappa-B kinase subunit gamma (IKBKG)] [21]. IKKβ is critical for the production of inflammatory cytokines and associated with the activation of cancers, including breast cancer, pancreatic cancer, melanoma and acute myeloid leukemia [22]. MiR-200b is identified as regulator of epithelial-mesenchymal transition (EMT), which is a process that accelerates tissue remodeling and gain a motile phenotype. The induction of EMT is regulated by several transcription factors, including ZEB1 and ZEB2, which negatively control E-cadherin and activate EMT [23–27]. To elucidate the mechanism how miR-200b regulates the expression of inflammatory cytokines, we transfected miR-200b expression plasmid or miR-200b inhibitor in human gingival fibroblasts (HGF) and examined the signaling pathways and the expressions of inflammatory cytokines.

## Materials and methods

### Reagents

Dulbecco’s modified Eagle’s medium (DMEM), ISOGEN II and human recombinant IL-1β were purchased from Wako (Tokyo, Japan). Human recombinant TNF-α was purchased from R&D Systems (Minneapolis, MN, USA). Fetal calf serum (FCS), Lipofectamine 2000, penicillin and streptomycin, and TrypLE™ Express were purchased from Invitrogen (Carlsbad, CA, USA). The PrimeScript RT reagent kit and SYBR Premix Ex Taq™ II were obtained from Takara (Tokyo, Japan). The miRNeasy Mini Kit was purchased from Qiagen (Valencia, CA, USA). The Mir-X miRNA First-Strand Synthesis Kit and SYBR Advantage qPCR Premix were purchased from Clontech (Mountain View, CA, USA). Expression plasmid for miRNA (pEZX-MR04) was obtained from GeneCopoeia (Rockville, MD, USA). miRCURY LNA inhibitor and miRCURY LNA inhibitor Control were purchased from Exiqon (Woburn, MA, USA). Anti-rabbit IgG (whole molecule)-peroxidase antibody produced in goat was from Sigma-Aldrich Japan (Tokyo, Japan). ELC plus Western Blotting Detection Reagents were purchased from GE Healthcare UK Ltd. (Buckinghamshire, UK). All chemicals used were of analytical grade.

### Cell cultures

HGF were cultured at 37 °C in 5% CO_2_ and 95% air in DMEM medium containing 10% FCS as described previously [6]. The Institutional Internal Review and Ethics Board at the Nihon University School of Dentistry at Matsudo approved the study (EC03-041, EC10-040, EC14-023). Written informed consent was obtained from each study subject after all procedures had been fully explained. The cells were grown to confluence in 100 mm cell culture dishes. Twenty-four hours after plating, cells at 40–60% confluence were transfected with control plasmid (pEZX-MR04; 3 μg) or miRExpress™ precursor miRNA expression plasmid for miR-200b (3 μg), miRCURY LNA inhibitor Control (10 nM) or miRCURY LNA inhibitor for miR-200b (10 nM) using a Lipofectamine 2000, then cultured for 12 h in DMEM without FCS and stimulated with IL-1β (1 ng/ml) or TNF-α (10 ng/ml). Total RNA was purified from triplicate cultures after stimulation for 24 h.

### Real-time polymerase chain reaction (PCR)

Total RNA (1 µg) was used as a template for cDNA synthesis. cDNA was prepared using the PrimeScript RT reagent kit. Quantitative real-time PCR was performed using the following primer sets: IL-6 forward; 5′-AAGCCAGAGCTGTGCAGATGAGTA-3′, IL-6 reverse; 5′-TGTCCTGCAGCCACTGGTTC-3′, IL-1β forward; 5′-CCAGGGACAGGATATGGAGCA-3′, IL-1β reverse; 5′-TTCAACACGCAGGACAGGTACAG-3′, IKKβ forward; 5′-ACTTGGCGCCCAATGACCT-3′, IKKβ reverse; 5′-CTCTGTTCTCCTTGCTGCA-3′, ZEB1 forward; 5′-CTTGAACGTCACATGACATCACATA-3′, ZEB1 reverse; 5′-TCTTGCAGTTTGGGCATTCATA-3′, CDH1 (E-cadherin) forward; 5′-AAGTGCTGCAGCCAAAGACAGA-3′, E-cadherin reverse; 5′-AAATTGCCAGGCTCAATGACAAG-3′, cyclooxygenase-2 (COX-2) forward; 5′-GAAGTACCAAGCTGTGCTTGAATAA-3′, COX-2 reverse; 5′-GGCTTGATTCCAATGCACCTA-3′, GAPDH forward; 5′-GCACCGTCAAGGCTGAGAAC-3′, GAPDH reverse; 5′-ATGGTGGTGAAGACGCCAGT-3′, using the SYBR Premix Ex Taq II in a TP800 thermal cycler dice real-time system (Takara, Tokyo, Japan). The amplification reactions were performed in a total volume of 25 µl 2 × SYBR Premix Ex Taq II (12.5 µl), 10 µM forward and reverse primers and 50 ng cDNA for IL-6, IL-1β, IKKβ, ZEB1, E-cadherin, COX-2 and 10 ng cDNA for GAPDH. For miRNA analysis, cDNA was synthesized by using the Mir-X miRNA First-Strand Synthesis kit. Quantitative real-time PCR was performed using SYBR Advantage qPCR Premix and the following primer sets: hsa-mir-200b forward primer; 5′-TAATACTGCCTGGTAATGATGA-3′, mRQ 3′ reverse primer, U6 forward primer and U6 reverse primer. The amplification reactions were performed in 25 µl of the final reaction mixture containing 2 × SYBR Advantage qPCR Premix (12.5 µl), 10 µM forward and reverse primers (final concentration, 0.2 µM) and 25 ng (2.0 µl) cDNA. To reduce variability between replicates, PCR premixes containing all reagents except for cDNA were prepared and aliquoted into 0.2 ml PCR tubes. The conditions for thermal cycling were 10 s at 95 °C, and 40 cycles of 5 s at 95 °C and 30 s at 60 °C. The expressions of IL-6, IL-1β, IKKβ, ZEB1, E-cadherin, COX-2 relative to GAPDH and miR-200b relative to U6 were determined in triplicate.

### Cytokine measurements

IL-6 in culture supernatants was measured with Human ELISA kits (Diaclone, Besançon Cedex, France) according to the manufacturer’s protocols.

### Western blotting

Radioimmunoprecipitation (RIPA) Lysis Buffer System (sc-24948; Santa Cruz, Paso Robles, CA, USA) was used for total protein extraction. Protein samples were separate on 10% sodium dodecyl sulfate polyacrylamide gel electrophoresis (SDS–PAGE) and transferred onto Hybond-P membrane. The membranes were then incubated for 3 h by anti-IKKβ (#2684; Cell Signaling Technology, Boston, USA), anti-ZEB1 (H-102; Santa Cruz, Paso Robles, CA, USA), anti-E-cadherin (#3195; Cell Signaling Technology, Boston, USA) and anti-α-tubulin antibody (sc-5286; Santa Cruz, Paso Robles, CA, USA) antibodies. Anti-rabbit and mouse IgG conjugated with HRP were used as the secondary antibodies. Immunoreactivities were determined by ECL plus Western Blotting Detection Reagents.

### miRNA target analysis

Potential miR-200b target mRNAs associated with inflammatory cytokines production were predicted using TargetScan (6.2) (http://www.targetscan.org) and microRNA.org website (http://www.microrna.org/). TargetScan is a database that predicts biological targets of miRNAs by searching for the presence of sites that match the seed region of each miRNA according to matches with the 7–8 bp seed region [28]. MicroRNA.org is a comprehensive resource of microRNA target predictions and expression profiles. Target predictions are based on a development of the miRanda algorithm which incorporates current biological knowledge on target rules and on the use of an up-to-date compendium of mammalian microRNAs [29, 30].

### Statistical analysis

Triplicate samples were analyzed for each experiment. Significant differences control and miR-200b and inflammatory cytokines treatments were analyzed using one-way ANOVA.

## Results

### miRNA-200b is induced by inflammatory cytokines in HGF

**Fig. 1:**
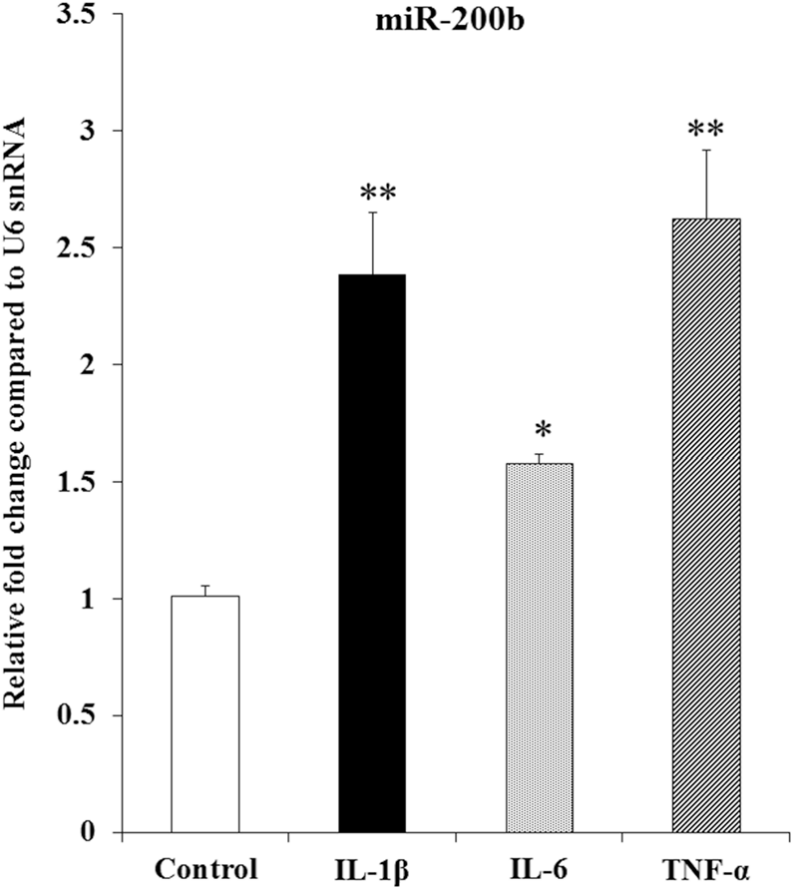
Inflammatory cytokines increased miR-200b expression in HGF. mRNA levels of miR-200b were determined by real-time PCR. U6 snRNA was used as an internal normalization control. HGF were stimulated by IL-1β (1 ng/ml), IL-6 (1 ng/ml) or TNF-α (10 ng/ml) for 24 h. Error bars indicate the ± standard deviation (SD) (*n* = 3). Significant differences versus control. *P < 0.05, **P < 0.01

We have previously demonstrated that the expression of miR-200b was increased in inflamed gingiva compared with non-inflamed gingiva using miRNA microarray [6]. To determine whether miR-200b is induced by inflammatory cytokines, we stimulated HGF by IL-1β (1 ng/ml), IL-6 (1 ng/ml) and TNF-α (10 ng/ml) for 24 h and revealed that they increased miR-200b expression in HGF (Fig. 1). These results suggest that miR-200b is associated with inflammatory diseases such as periodontitis.

### miR-200b regulates the expression of IL-6 and IL-1β in HGF

**Fig. 2:**
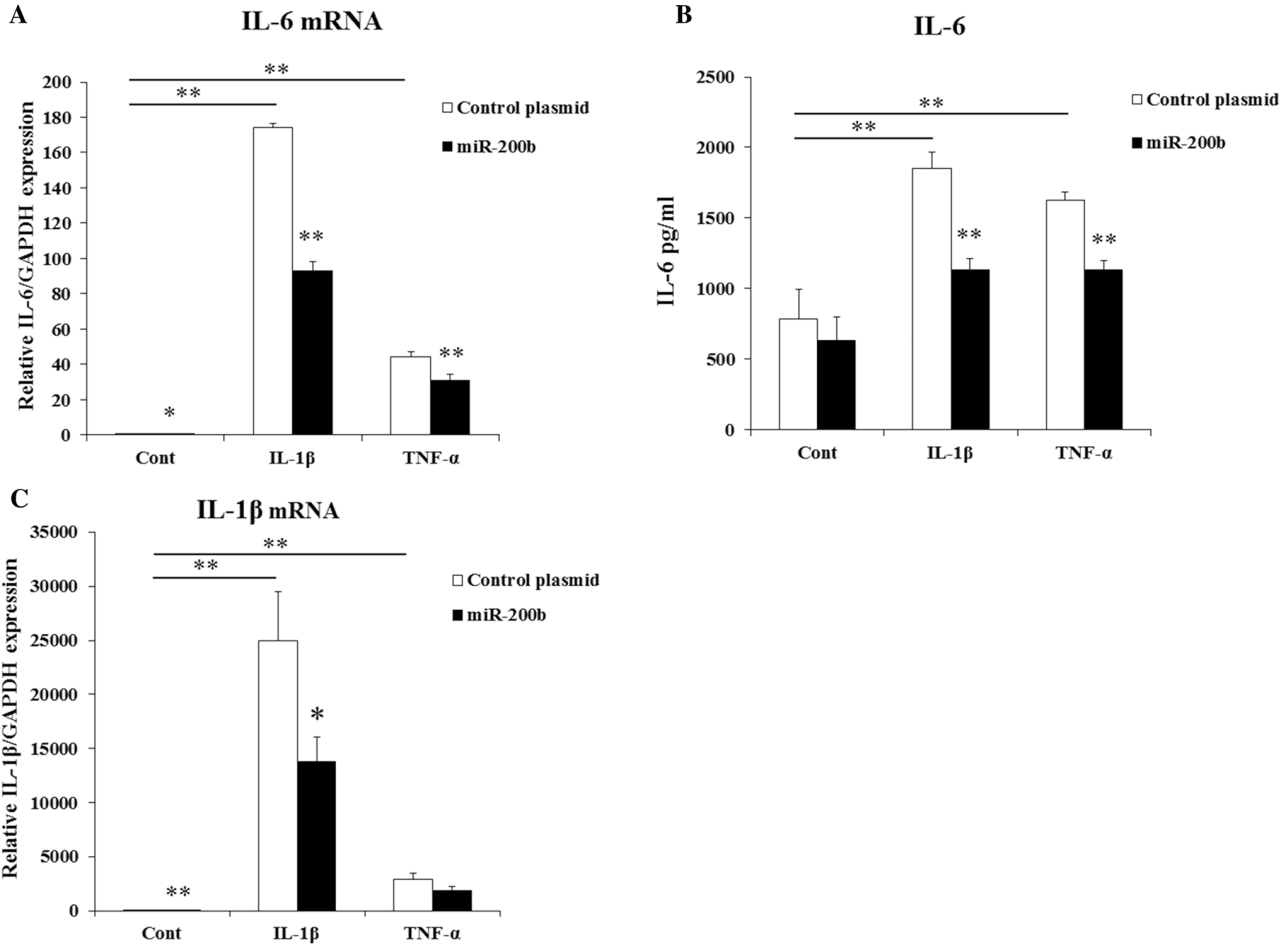
Effects of miR-200b on the expressions of IL-6 and IL-1β in HGF. IL-6 (**a**) and IL-1β (**c**) mRNA levels in HGF were measured by real-time PCR. GAPDH was used as an internal normalization control. IL-6 (**b**) protein expressions were determined by ELISA. HGF were transfected with miR-200b expression plasmid or control plasmid and treated with IL-1β (1 ng/ml) or TNF-α (10 ng/ml) for 24 h. Quantitative analyses of the datasets (*n* = 3) are shown with SD. Significant differences from control. *P < 0.05, **P < 0.01

To determine the effects of miR-200b on the expression of IL-6 and IL-1β, HGF were stimulated by IL-1β (1 ng/ml) or TNF-α (10 ng/ml) and transfected with miR-200b expression plasmid or control plasmid. IL-1β and TNF-α induced mRNA and protein levels of IL-6 and mRNA levels of IL-1β, but they were significantly suppressed by miR-200b overexpression in HGF (Fig. 2a–c). In addition, miR-200b decreased mRNA expression of IL-6 and IL-1β without stimulation by IL-1β or TNF-α (Fig. 2a, c).

### miR-200b inhibition increases IL-6 production

**Fig. 3:**
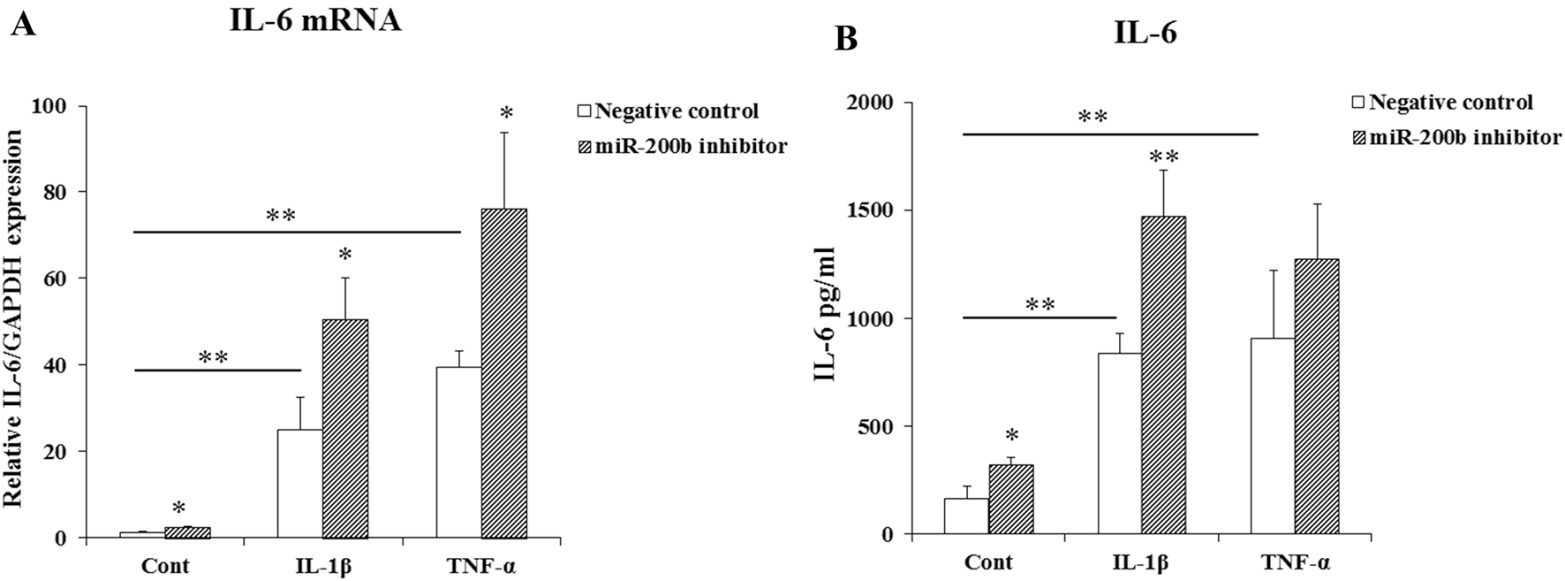
Effects of miR-200b inhibitor on the expression of IL-6 in HGF. IL-6 mRNA (**a**) and protein levels (**b**) in HGF was measured by real-time PCR and ELISA. HGF were transfected with miR-200b inhibitor (10 nM) or negative control and stimulated by IL-1β (1 ng/ml) or TNF-α (10 ng/ml) for 24 h. Quantitative analyses of the datasets (*n* = 3) are shown with SD. Significant differences from control. *P < 0.05, **P < 0.01

We used miR-200b inhibitor or negative control oligonucleotide (10 nM) to determine if miR-200b inhibition could lead to changes in IL-6 production. HGF were transfected with miR-200b inhibitor or negative control oligonucleotide and stimulated by IL-1β or TNF-α for 24 h. IL-1β and TNF-α up-regulated IL-6 mRNA and protein levels in HGF and they were further significantly increased by miR-200b inhibitor (Fig. 3a, b).

### miR-200b targets IKKβ, ZEB1, E-cadherin and COX-2

**Fig. 4:**
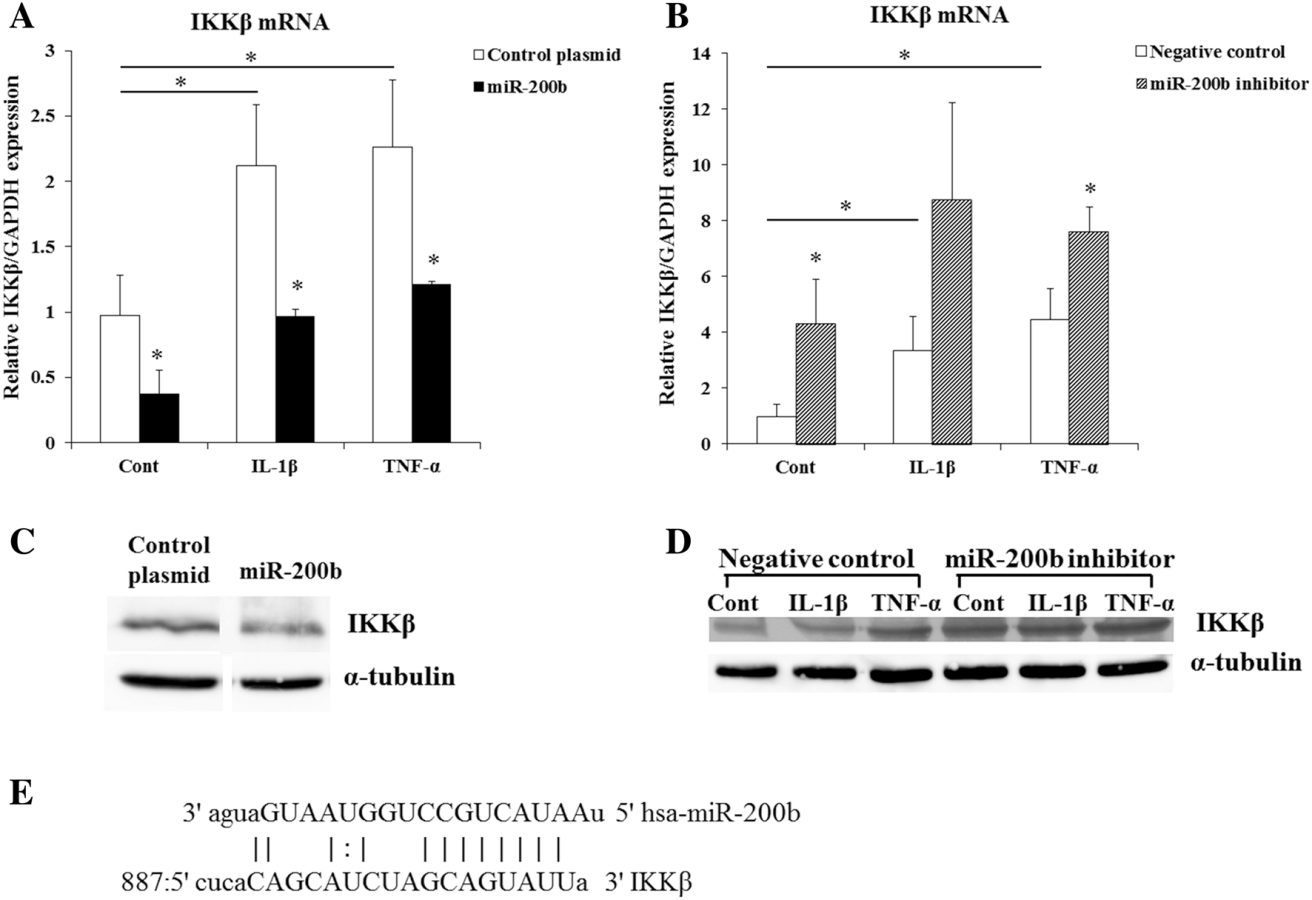
IKKβ is a putative target of miR-200b. IKKβ mRNA levels (**a, b**) in HGF were measured by real-time PCR. GAPDH was used as an internal normalization control. IKKβ protein levels (**c, d**) were determined by Western blotting. HGF were transfected with miR-200b expression plasmid or miR-200b inhibitor and treated with or without IL-1β (1 ng/ml) or TNF-α (10 ng/ml) for 24 h. IKKβ 3′-UTR has a putative miR-200b seed site (**e**). Quantitative analyses of the datasets (*n* = 3) are shown with SD. Significant differences from control. *P < 0.05

To elucidate how miR-200b regulates IL-6 production, we used miRanda and TargetScanHuman (6.2) to predict the target genes of miR-200b and found a putative miR-200b binding site on the 3′-UTR of IKKβ (Fig. 4e). IKKβ is a subunit of IKK and essential for the activation of NF-κB [21, 22]. To confirm the miR-200b regulates IKKβ expression, HGF were stimulated by IL-1β or TNF-α and transfected with miR-200b expression plasmid or miR-200b inhibitor, and examined IKKβ mRNA and protein levels. IL-1β and TNF-α significantly induced IKKβ mRNA and protein levels in HGF. MiR-200b overexpression suppressed and miR-200b inhibitor increased the mRNA and protein levels of IKKβ (Fig. 4a–d).

**Fig. 5:**
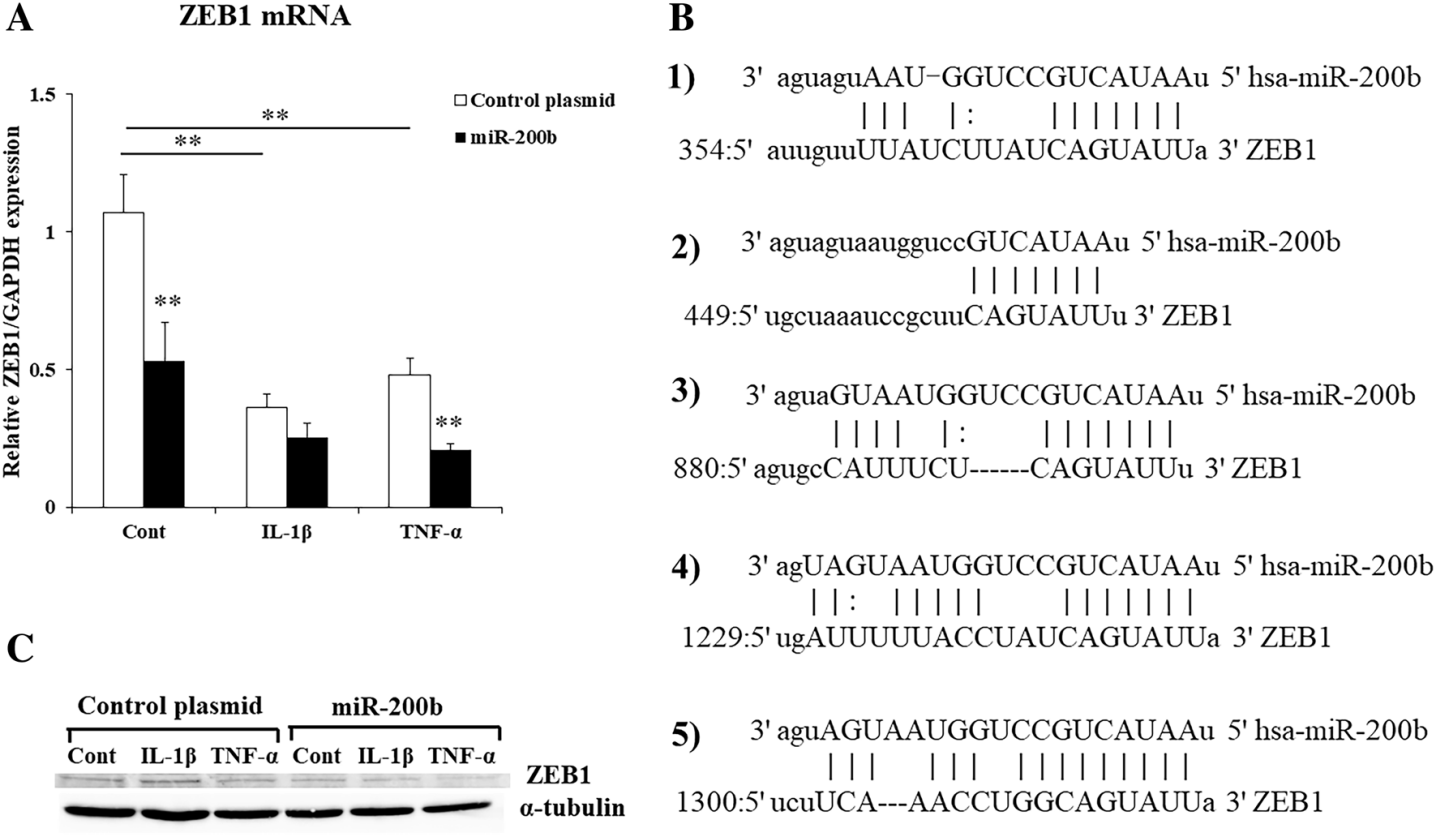
miR-200b targets ZEB1. ZEB1 mRNA levels (**a**) in HGF were measured by real-time PCR. GAPDH was used as an internal normalization control. ZEB1 protein levels (**c**) were determined by Western blotting. HGF were transfected with miR-200b expression plasmid or control plasmid, and treated with or without IL-1β (1 ng/ml) or TNF-α (10 ng/ml) for 24 h. miRanda and TargetScanHuman (6.2) showed that Zeb1 3′-UTR contains several putative miR-200b target sites (**b1**–**5**)

**Fig. 6:**
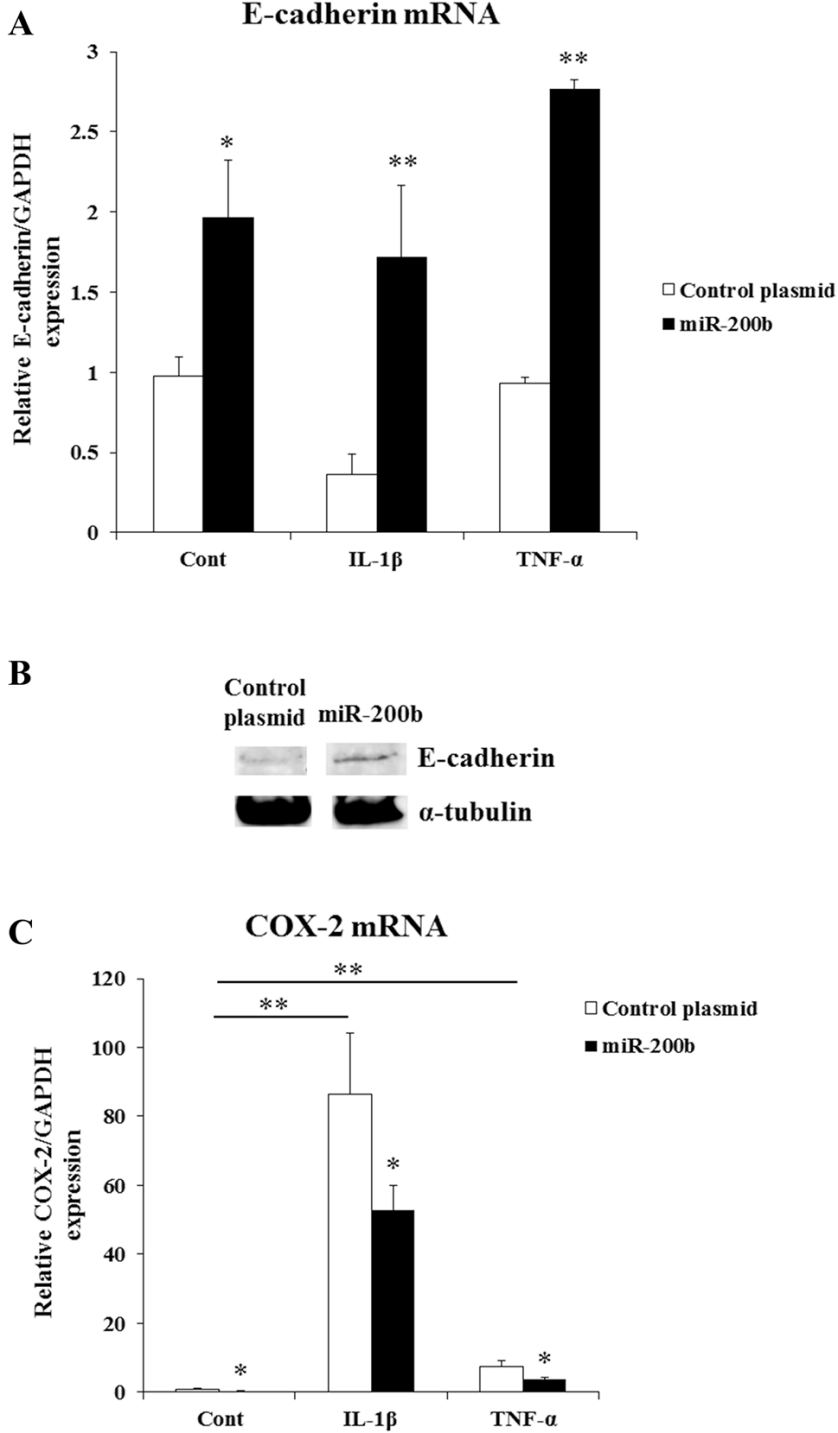
Effects of miR-200b on the expressions of E-cadherin and COX-2 in HGF. E-cadherin (**a**) and COX-2 (**c**) mRNA levels in HGF were determined by real-time PCR. GAPDH was used as an internal normalization control. E-cadherin protein levels (**b**) were assayed by Western blotting. HGF were transfected with miR-200b expression plasmid or control plasmid, and stimulated with or without IL-1β (1 ng/ml) or TNF-α (10 ng/ml) for 24 h. Quantitative analyses of the datasets (*n* = 3) are shown with SD. Significant differences from control. *P < 0.05, **P < 0.01

ZEB1 3′-UTR contains several putative miR-200b target sites (Fig. 5b1–5). HGF were stimulated by IL-1β or TNF-α and transfected with miR-200b expression plasmid or control plasmid. IL-1β and TNF-α significantly inhibited ZEB1 mRNA levels in HGF, and they were further suppressed by miR-200b overexpression (Fig. 5a). MiR-200b repressed ZEB1 protein levels in HGF (Fig. 5c). In addition, E-cadherin mRNA and protein levels were increased by miR-200b overexpression in HGF (Fig. 6a, b). We showed COX-2 is another target of miR-200b and miR-200b overexpression suppressed COX-2 mRNA levels in FGF (Fig. 6c).

## Discussion

miRNAs are novel epigenetic factors controlling gene expression at the post-transcriptional level through imperfect base pairing with the 3′-UTRs of target mRNAs. Several miRNAs have been identified as important transcriptional regulators of inflammation and functions of microRNAs have been proposed in the regulation of innate immune responses [31–33]. The innate immune response plays an important role in the defense against pathogens. It can be initiated by binding of microbial ligands to membrane-associated pathogen recognition receptor known as Toll-like receptors (TLRs). The members of miR-200 family have been shown to regulate innate immune response by targeting the TLR4 signaling pathway via MyD88 which is essential for mediating signals through TLR [34]. Recent studies demonstrated that miR-200b decreased inflammatory response of activated microglia by inhibiting c-Jun/MAPK pathway [35], and miR-200c regulated IL-8 expression by targeting IKKβ [36]. miR-200b and miR-200c are also known to control EMT via ZEB1 [23, 27]. EMT is a complex process through which epithelial cells lose intercellular adhesion, acquire mesenchymal phenotype and increase migratory and invasive properties. ZEB1 in the progression of breast cancer controlled the production of IL-6 and IL-8, along with the initiation of EMT [37]. ZEB1 downregulates E-cadherin and activate EMT. It has been shown that E-cadherin negatively controls the transcriptional activity of NF-κB to associate with the p65 subunit of NF-κB heterodimer, leading to p65 cytoplasmic sequestration [20, 38]. In this study, ZEB1 mRNA and protein levels were suppressed by miR-200b overexpression (Fig. 5a, c). In addition, miR-200b increased E-cadherin mRNA and protein expressions in HGF (Fig. 6a, b). These results suggest that miR-200b controls E-cadherin expression by targeting ZEB1 and might modulate the transcriptional activity of NF-κB via E-cadherin.

NF-κB is a nuclear factor that regulates various cellular processes, including survival, proliferation, immune responses and inflammation. NF-κB signaling is composed of two pathways, the canonical pathway and the non-canonical pathway. IL-1β and TNF-α induce the canonical pathway [20]. For IL-1-mediated signaling, it has been shown that the polyubiquitination of TNF receptor-associated factor 6 (TRAF6) leads to the activation of TGF-beta-activated kinase 1 (TAK1) which phosphorylates and activates IKKβ [39–41]. For the TNFα-induced signaling pathway, TRAF2 and TRAF5 catalyze K63-polyubiquitination of receptor-interacting protein-1 (RIP1) and IKKγ/NEMO, which contributes to the activation of TAK1. Activation of IKKβ was induced by phosphorylation of serine 177 and 181 via TAK1 [41, 42]. In the canonical pathway, IKKβ is necessary and sufficient for phosphorylation of IκBα, which leads to the activation of NF-κB and associated with prevention of apoptosis, cytokine production and inflammation. The deletion of the IKK-regulatory subunit IKKγ/NEMO in hepatocytes prevents NF-κB activation and triggers spontaneous liver apoptosis, chronic hepatitis and the development of liver fibrosis and hepatocellular carcinoma [43]. In addition, it has been shown that IKKβ activated NF-κB and induced IL-6 production [44]. In this study, miR-200b decreased IKKβ mRNA and protein expressions (Fig. 4a, c). Conversely, miR-200b inhibitor increased IKKβ mRNA and protein levels (Fig. 4b, d). These results suggest that miR-200b regulates the expression of IL-6 and IL-1β via IKKβ.

IL-6 is a multifunctional cytokine that regulates cell proliferation, survival, immune and inflammatory response. IL-6 gene is induced in response to bacterial endotoxin (LPS), or a variety of other cytokines such as TNF-α and IL-1β. IL-6 is also known to play major roles in the pathogenesis of periodontitis. Fibroblasts constitutively express a low level of IL-6; in addition, IL-1 and TNF-α synergistically enhance IL-6 production in the fibroblasts [45–47]. IL-1β and TNF-α induced NF-κB expression, and then NF-κB controlled the expression of inflammatory cytokines, such as IL-1β, IL-6 and TNF-α [48–50].

In this study, we found that miR-200b attenuated the expressions of IL-6 and IL-1β in HGF. We also have shown that miR-200b targets IKKβ and ZEB1 which plays a key role in the production of inflammatory cytokines and modulates the expression of E-cadherin via ZEB1. E-cadherin is critical for regulating EMT involved in various developmental processes including the mesoderm formation and the neurulation. In addition, E-cadherin negatively controls the NF-κB activity to associate with the p65 subunit of NF-κB heterodimer, leading to p65 cytoplasmic sequestration. These results suggest that miR-200b attenuates inflammatory cytokines such as IL-6 and IL-1β productions mediated through IKKβ and ZEB1 using a negative feedback loop between miR-200b and the NF-κB pathway in the inflammed gingiva.
